# Queuine Salvaging in the Human Parasite *Entamoeba histolytica*

**DOI:** 10.3390/cells11162509

**Published:** 2022-08-12

**Authors:** Lotem Sarid, Jingjing Sun, Jurairat Chittrakanwong, Meirav Trebicz-Geffen, Jun Ye, Peter C. Dedon, Serge Ankri

**Affiliations:** 1 Department of Molecular Microbiology, Ruth and Bruce Rappaport Faculty of Medicine, Technion, Haifa 31096, Israel; 2 Department of Biological Engineering, Massachusetts Institute of Technology, Cambridge, MA 02139, USA; 3 Applied Biological Sciences Program, Chulabhorn Graduate Institute, Chulabhorn Royal Academy, Bangkok 10210, Thailand

**Keywords:** queuine, queuosine, tRNA modifications, *Entamoeba histolytica*, oxidative stress resistance

## Abstract

Queuosine (Q) is a naturally occurring modified nucleoside that occurs in the first position of transfer RNA anticodons such as Asp, Asn, His, and Tyr. As eukaryotes lack pathways to synthesize queuine, the Q nucleobase, they must obtain it from their diet or gut microbiota. Previously, we described the effects of queuine on the physiology of the eukaryotic parasite *Entamoeba histolytica* and characterized the enzyme EhTGT responsible for queuine incorporation into tRNA. At present, it is unknown how *E. histolytica* salvages queuine from gut bacteria. We used liquid chromatography–mass spectrometry (LC–MS) and N-acryloyl-3-aminophenylboronic acid (APB) PAGE analysis to demonstrate that *E. histolytica* trophozoites can salvage queuine from Q or *E. coli* K12 but not from the modified *E. coli* QueC strain, which cannot produce queuine. We then examined the role of EhDUF2419, a protein with homology to DNA glycosylase, as a queuine salvage enzyme in *E. histolytica*. We found that glutathione S-transferase (GST)-EhDUF2419 catalyzed the conversion of Q into queuine. Trophozoites silenced for EhDUF2419 expression are impaired in their ability to form Q-tRNA from Q or from *E. coli*. We also observed that Q or *E. coli* K12 partially protects control trophozoites from oxidative stress (OS), but not siEhDUF2419 trophozoites. Overall, our data reveal that EhDUF2419 is central for the direct salvaging of queuine from bacteria and for the resistance of the parasite to OS.

## 1. Introduction

In many parts of the world, poor sanitation and unsafe hygiene practices are causing amebiasis to spread. The World Health Organization estimates that 50 million people in India, Southeast Asia, Africa, and Latin America suffer from amebic dysentery and amebiasis each year, resulting in at least 100,000 deaths. Amebiasis is primarily transmitted through ingesting contaminated food or water containing *E. histolytica* cysts. After the cyst form has been swallowed by the host, excystation occurs in the intestinal lumen, followed by colonization of the large intestine by the trophozoites. In this next divide and encyst; both trophozoites and cysts are excreted in stools. *E. histolytica* trophozoites reside in the colon as a non-pathogenic commensal in most infected individuals (90% of infected individuals are asymptomatic). For unknown reasons, the trophozoites can become virulent and invasive, cause amebic dysentery, and migrate to the liver via the portal veins, where they cause hepatocellular damage. No vaccine against amebiasis currently exists; the drug of choice for treating amebiasis is metronidazole, which, however, may have severe side effects. Additionally, some clinical strains of *E. histolytica* are less sensitive to metronidazole, suggesting the emergence of metronidazole-resistant strains [1]. In recent years, RNA modifications are emerging as an essential means to maintain the cell life cycle in numerous contexts, ranging from infectious disease to neuropathology [2] and cancer [3]. More than 100 RNA chemical modifications are known to date, addressing all RNA species. Nevertheless, RNA-modifying enzymes have not yet been exploited as drug targets.

Queuosine (Q) and its glycosylated derivatives occur in position 34 of the anticodon of tRNA with G_34_U_35_N_36_ in the anti-codon loop of eubacteria and eukaryotes except for *Saccharomyces cerevisiae* [4,5]. Q is highly conserved and found in bacteria, plants, fishes, insects, and mammals. While bacteria can synthesize queuine (the nucleobase of Q) *de novo*, salvage of the prokaryotic Q precursors preQ_0_ and preQ_1_ has recently been reported [6]. Eukaryotes are not capable of Q synthesis and rely on the salvage of queuine as a Q precursor either by nutrition or by intestinal bacterial flora [7,8,9]. The tRNA-guanine transglycosylase (TGT) is the main enzyme responsible for the incorporation of Q into tRNA in place of G34. The cyclopentendiol moiety is synthesized at the level of tRNA from unknown precursors and enzymes in both eubacterial and eukaryotic species. The crystal structure of hTGT in its heterodimeric form and in complex with a 25-mer stem-loop RNA has been recently established [10]. The detailed analysis of its dimer interface and interaction with a minimal substrate RNA indicates that one base only, guanine 34 or queuine, can simultaneously reside at the active site in support of a “ping-pong” mechanism that has already been proposed for *E. coli* TGT [11]. Regarding hQTRTD1, the authors proposed that it could serve to anchor the TGT enzyme in the compartmentalized eukaryotic cell [10]. Based on the annotation of the *E. histolytica* genome, a homolog of *h*QTRT1 and *h*QTRTD1 exists in *E. histolytica*, namely, *Eh*QTRT1 (XP_656142.1) and *Eh*QTRTD1 (XP_652881.1). Our previous work has significantly contributed to our understanding of *E. histolytica* tRNA-guanine transglycosylase (TGT) (EhTGT) and the regulation of *E. histolytica*’s virulence by queuine [12]. We found that EhTGT is a heterodimer composed of *Eh*QTRT1 and *Eh*QTRTD1. EhTGT is catalytically active, and it incorporates queuine into *E. histolytica* tRNAs. The presence of Q in tRNA^Asp^_GUC_ stimulates its methylation by Ehmeth, a Dnmt2-type multisubstrate tRNA methyltransferase, at the C38 position. Queuine does not affect the growth of the parasite, it protects the parasite against oxidative stress (OS), and it antagonizes the negative effect that OS has on translation by inducing the expression of genes involved in OS response, such as heat shock protein 70, antioxidant enzymes, and enzymes involved in DNA repair. On the other hand, queuine impairs *E. histolytica* virulence determined in a mouse model of amebic colitis by downregulating the expression of genes previously associated with virulence, including cysteine proteases, cytoskeletal proteins, and small GTPases [12]. Silencing of EhQTRT1 expression prevents the incorporation of queuine into tRNAs and impairs the methylation of C38 in tRNA^Asp^_GUC_, which inhibits the growth of the parasite, impairs its resistance to OS and its cytopathic activity.

Information about how queuine is salvaged by eukaryotic organisms is scanty. In mammalian cells, queuine is generated from Q-5′-phosphate, which suggests that it originated from degraded tRNA during the normal turnover process [13]. In the green algae *Chlorella pyrenoidosa* and *Chlamydomonas reinhardtii*, an enzymatic activity of unknown nature that salvages queuine from Q has been identified [14]. A search of genes that co-distribute with eukaryotic QTRT1 and QTRTD1 identified a potential protein, DUF2419, that salvages queuine from Q [15]. The structural similarity of DUF2419 with DNA glycosylases suggests a ribonucleoside hydrolase activity. Indeed, genetic evidences support the role of DUF2419 as an enzyme that salvages queuine from Q in *Schizosaccharomyces pombe*, humans, maize, and *Streptococcus thermophilus* [15]. Here, we used a genetic approach coupled with LC-MS and APB PAGE analysis to demonstrate that EhDUF2419 is the enzyme that salvages queuine from Q or from *E. coli* K12 in *E. histolytica*.

## 2. Materials and Methods

### 2.1. E. histolytica Culture 

*E. histolytica* trophozoites, the HM-1:IMSS strain (a gift from Prof. Samudrala Gourinath, Jawaharlal Nehru University, New Delhi, India), were grown at 37 °C in 13 × 100 mm screw-capped Pyrex glass tubes or plastic culture flasks in TYI-S-33 medium to exponential phase. Trophozoites were harvested from their growth support by incubating the tubes or flasks in an ice-water bath for 5 min followed by centrifugation according to a previously reported protocol [16]. In some experiments, the trophozoites were cultivated with Q (0.1 μM) (a gift from Prof. Peter C. Dedon, MIT, USA) or queuine (0.1 μM) (a gift from Prof. Hans-Dieter Gerber and Prof. Klaus Reuter, University of Marburg, Marburg, Germany) in TYI-S-33 medium for three days at 37 °C and then harvested for further analysis as described before. 

### 2.2. Transfection of E. histolytica Trophozoites

The transfection of *E. histolytica* trophozoites was performed using a previously described protocol [17]. Around 10^5^ trophozoites were seeded onto 35 mm diameter wells of a 6-well culture plate and cultivated in 9 mL of TYI-S-33 medium at 37 °C for 15 h in an anaerobic jar. The LipofectAMINE-plasmid DNA complexes were prepared in OPTI-MEM I medium (Life Technologies, Rhenium, Modi’in, Israel) supplemented with 5 mg/mL cysteine and 1 mg/mL ascorbic acid (transfection medium). To silence EhDUF2419 (siEhDUF2419 vector), 30 μL of the transfection medium containing 4 μg of vector used was mixed with 15 μL of LipofectAMINE PLUS (Life Technologies) and kept at room temperature for 15 min. This mixture was combined with 20 μg (10 μL) of LipofectAMINE, kept at room temperature for 15 min, diluted with 945 μL of transfection medium, and added to the seeded trophozoites after removing TYI-S-33 medium. The plate was then incubated at 37 °C for 2.5 h. The trophozoites were transferred to fresh medium and further cultivated at 37 °C for 18 h. Next, 3 μg/mL G418 was added to the cultures for the selection of trophozoites that carry the siEhDUF2419 vector. Finally, transfected trophozoites were maintained in TYI-S-33 medium containing 6 μg/mL G418.

### 2.3. Resistance of E. histolytica Trophozoites to OS

Resistance to OS of trophozoites was determined by the eosin dye exclusion method [18]. Briefly, *E*. *histolytica* trophozoites (1 × 10^6^) were exposed to 2.5 mM H_2_O_2_ for 30 min at 37 °C. At the end of the exposure, a 10 μL aliquot of each culture was stained with eosin (0.1% final concentration), and the number of living trophozoites was counted in a counting chamber under a light microscope.

### 2.4. Growth Rate of E. histolytica Trophozoites

A size of 4 × 10^4^ *E. histolytica* trophozoites were grown in 15 mL tube in TYI-S-33 medium at 37 °C. The number of viable trophozoites was counted according to previously described protocol [18] after between 24 and 48 h of culture. 

### 2.5. Construction of GST-Tagged EhDUF2419 Vector

For construction of the pGEX-EhDUF2419 vector, EhDUF2419 was amplified from *E. histolytica* genomic DNA with the primers 5′ BamHI EhDUF2419 and 3′ EhDUF2419 (Table 1). The PCR product was cloned in a pGEM-T easy vector (Promega, IMBH, Beit Haemek, Israel), digested with BamHI and NotI, and then subcloned into a pGEX-4T-1 (Cytiva, Sigma-Aldrich, Rehovot, Israel) vector to generate pGEX-EhDUF2419 vector.

### 2.6. Construction of Silenced EhDUF2419 Vector

For construction of the siEhDUF2419 vector, EhDUF2419 was amplified from *E. histolytica* genomic DNA with the primers 5′ BglII EhDUF2419 and 3′ XhoI EhDUF2419 (Table 1). The PCR product was cloned in a pGEM-T easy vector (Promega, IMBH Beit Haemek, Israel), digested with BglII and XhoI, and then subcloned into a pEhEx-04-trigger vector containing a 142 bp trigger region (EHI_048660) (a kind gift of Tomoyoshi Nozaki, University of Tokyo, Japan) to generate siEhDUF2419 vector.

### 2.7. Preparation of Recombinant GST-Tagged EhDUF2419

Recombinant EhDUF2419 was expressed as GST-tagged protein in *E. coli* BL21(DE3)pLysS competent cells, which were transfected with pGEX vector-derived plasmids. The overnight culture was supplemented with ampicillin (100 µg/mL) and grown at 37 °C until the OD_600_ reached ~0.4. Synthesis of the GST-tagged protein complex was initiated by adding IPTG to the culture at a final concentration of 0.1 mM. After overnight incubation in the presence of IPTG at 16 °C, the bacteria were harvested and lysed with lysis buffer (100 mM KCl, 1 mM DTT, 1 mM PMSF, 0.1 µg/mL Lysozyme, 0.1 µg/mL Leupeptine, PBS) and set in ice for 30 min. The cells were sonicated on a Sonics Vibracell VCX750 Ultrasonic Cell Disrupter (Labotal Mevaseret Zion, Israel) and BugBuster protein extraction reagent (1:100 ratio) (Novagen, Mercury, Rosh Haayin, Israel) were added to complete the lysis. GST-tagged proteins were purified under native conditions on gluthatione-agarose resin (Sigma-Aldrich, Rehovot, Israel). The recombinant proteins were then washed 3 times with Buffer A (100 mM KCl, 1% Triton, 1 mM PMSF, PBS) and then 3 times with Buffer B (100 mM KCl, 1 mM PMSF, PBS). Next, the proteins were eluted with glutathione elution buffer (Tris-HCl 50 mM pH 9.6, L-glutathione reduced (Sigma-Aldrich, Rehovot, Israel) 10 mM, 150 mM NaCl). Eluted proteins were resolved on 12% SDS gel and the gel was stained with silver stain (Pierce-Thermofisher, Ornat, Ness Ziona, Israel).

### 2.8. Preparation of Recombinant GST

Recombinant GST was expressed in *E. coli* BL21(DE3)pLysS competent cells, which were transfected with pGEX-4T-1 vector. The proteins were prepared according to the protocol described above.

### 2.9. Enzymatic Activity of EhDUF2419

Twenty-five µg of recombinant GST or GST-tagged EhDUF2419 was incubated with 409 ng of Q in 30 μL HEPES-reaction buffer (100 mM HEPES pH 7.3, 20 mM MgCl_2_, 5 mM DTT) at 37 °C or at room temperature overnight. Next, GST or GST-EhDUF2419 was pulldown from the reaction with glutathione-agarose resin (Sigma-Aldrich, Rehovot, Israel). The level of Q vs. queuine in the samples was determined by LC-MS/MS as described below.

### 2.10. Quantification of tRNA Modifications in E. histolytica by LC-MS/MS

The method for tRNA modification quantification was modified from an established LC-MS/MS method, which includes tRNA purification, tRNA hydrolyzation, and LC-MS/MS analysis [19]. 

### 2.11. tRNA Purification Using HPLC

Total RNA was extracted from *E. histolytica* trophozoites that were incubated with *E. coli* K12/∆QueC (a kind gift of Prof. Valérie de Crécy-Lagard, University of Florida, Gainesville, FL, USA) using the Monarch Total RNA Miniprep Kit (NEW ENGLAND BioLabs, Ornat, Nes Ziona, Israel). According to the manufacturer’s instructions, the absence of mechanical disruption during the cell lysis step favors the extraction of *E. histolytica* RNA over *E. coli* RNA using the detergent-based lysis buffer. tRNA samples were purified by high-performance liquid chromatography (1200 Infinity, Agilent, Santa Clara, CA, USA) using a size exclusion column (300 × 7.8 mm, 300 Å, Agilent, Santa Clara, CA, USA) with an isocratic mobile phase (100 mM ammonium acetate, pH 7.6, 40 °C) at a flow rate of 1 mL/min. tRNA fractions were collected and further dried by SpeedVac vacuum concentrators. These tRNA samples were reconstituted in RNase-free water, measured on a Nanodrop spectrophotometer 2000 (Thermo Fisher Scientific, Waltham, MA, USA) and quality checked on a bioanalyzer nano chip (Agilent, Santa Clara, CA, USA). 

### 2.12. Hydrolysis of tRNA to Nucleosides

Purified tRNA samples (400 ng) were hydrolyzed in a reaction mixture (30 µL) containing 2.5 mM MgSO_4_, 5 mM Tris-HCl (pH 8.0), 0.1 µg/mL coformycin, 0.1 mM deferoxamine, 0.1 mM butylated hydroxytoluene, 0.083 U/µL benzonase, 0.1 U/µL calf intestinal alkaline phosphatase, 0.003 U/µL phosphodiesterase I and 50 nM internal standard [^15^N]_5_-deoxyadenosine. The reactions were incubated at 37 °C for 6 h and used for LC-MS/MS analysis without further purification.

### 2.13. LC-MS/MS Quantification Analysis

LC-MS/MS analysis method was developed and optimized by using synthetic standards to achieve maximal sensitivity for target detection, including the LC gradient and the retention time, m/z of the transmitted parent ion, *m*/*z* of the monitored product ion, fragmentor voltage, and collision energy. The ribonucleosides were resolved a Synergi Fusion-RP C18 column (100 × 2.0 mm, 2.5 µm, Phenomenex, Torrance, CA, USA) with a gradient starting with 100% phase A (5 mM ammonium acetate, pH 5.3), followed by 0–10% phase B (acetonitrile) 0–10 min; 10–40% phase B, 10–14 min; 40–80% phase B, 14–15 min; 80–90% phase B, 15–15.1 min; 90% phase B, 15.1–18 min at 35 °C and a flow rate of 0.35 mL/min. The HPLC column was coupled to an Agilent 6490 Triple Quad mass spectrometer with an electrospray ionization source in positive mode with the following parameters: gas temperature, 200 °C; gas flow, 11 L/min; nebulizer, 20 psi; sheath gas temperature, 300 °C; sheath gas flow, 12 L/min; capillary voltage, 1800 V. MRM mode was used for detection of product ions derived from the precursor ions for all the modified ribonuleosides. Quantitative analysis was performed by normalizing MS signals by UV signals of canonical ribonuleosides.

### 2.14. N-Acryloyl-3-Aminophenylboronic Acid (APB) Northern Blotting for E. histolytica tRNA^His^_GUG_

APB gels were prepared and run with a few modifications according to Igloi and Kössel [20]. Briefly, 14 μg of total RNA extracted from *E. histolytica* using the TRI reagent kit according to the manufacturer’s instructions (Sigma-Aldrich, Rehovot, Israel) was deacetylated in 100 mM Tris-HCl (pH 9.0) for 30 min at 37 °C. RNA was ethanol precipitated and resuspended in 10 μL DEPC-treated water (IMBH, Beit-Haemek, Israel). Samples were then denatured for 10 min at 70 °C and run at 4 °C on Tris-acetate EDTA (TAE) buffer, 8 M urea, 15% acrylamide, and 5 mg/mL aminophenylboronic acid (Sigma-Aldrich, Rehovot, Israel) on Bio-Rad mini gels. The gel was run at 4 °C at 75 V for 7–8 h until the bromophenol blue reached the bottom of the gel. The gels were then stained with ethidium bromide in 1× TAE buffer for 20 min and then visualized for equal loading of samples. The gels were destained with ultrapure water for 20 min, and samples were transferred to a Nylon Hybridization Transfer membrane (PerkinElmer, Ra’anana, Israel) by electrotransfer using 0.5× TAE as the transfer buffer for 45 min at 130 mA. The membrane was cross-linked by UV using 120 mJ (Stratagene UV linker) and hybridized twice for 15 min each in 5 mL hybridization buffer (20 mM sodium phosphate buffer (pH 7), 300 mM NaCl, 1% SDS), followed by the addition of 150 μg/mL heat-denatured salmon sperm DNA (ssDNA) to the hybridization buffer and blocking for 1 h at 60 °C. The membrane was then incubated with 15 pmol\mL of biotinylated tRNA probes prepared against *E. histolytica* tRNA^His^_GUG_ and incubated at 60 °C for 16–18 h. The membrane was then washed for 10 min with 5 mL wash buffer (20 mM sodium phosphate buffer (pH 7), 300 mM NaCl, 2 mM EDTA, 0.5% SDS) at 60 °C and then incubated in hybridization buffer once at room temperature for 10 min. The membrane was then incubated in streptavidin-horseradish peroxidase (HRP) (GenScript, A2S, Yavne, Israel) conjugate in 5 mL hybridization buffer (1:5000) for 1 h followed by two washes for 10 min. The membranes were incubated with enhanced chemiluminescence reagent (Advansta, Bioconsult, Jerusalem, Israel), and exposed to a FUSION FX7 Western blot and chemiluminescence imaging system (Vilber, Marne-la-Vallée, France). 

### 2.15. Quantitative-Real Time PCR

Total RNA was extracted from control (WT) or siEhDUF2419 trophozoites using TRI reagent (Sigma-Aldrich, Rehovot, Israel) and the amount of total RNA was quantified using nanodrop spectrophotometer (ThermoFisher scientific, Bargal, Shoham, Israel). Reverse transcription was performed using the RevertAid First strand cDNA synthesis kit (Thermofisher, Rhenium, Modi‘in, Israel), according to the manufacturer’s instructions. Primers used to amplify EhDUF2419 (5′EhDUF2419 set3 and 3′EhDUF2419 set3) and rDNA (rDNA5′ and rDNA 3′) are described in Table 1. qRT-PCR was performed using the qPCRBio SyGreen Mix Hi-ROX (PCR biosystems, Tamar, Abu-Gosh, Israel) according to the manufacturer’s instructions and run on the Real-Time PCR QuantStudio3 (Thermofisher, Qiryat Shemona, Israel) using the following conditions: Initial denaturation step at 95 °C for 2 min, 40 cycles of denaturation at 95 °C for 5 s, followed by hybridization at 50 °C for 30 s. The melting curve was performed according to the following conditions: 95 °C for 15 s, then 60 °C for 1 min, and finally at 95 °C for 15 s. The relative fold change was calculated using the 2^^(−ΔΔCt)^ method [21]. The QRT-PCR values were normalized to the level of rDNA [22]. PCR amplification controls were performed for each primer to verify the formation of a single PCR product.

### 2.16. Western Blotting

Western blotting of total protein extract of *E. histolytica* trophozoites (40 µg) was performed according to previously described protocol [12]. Briefly, the proteins were resolved on 12% SDS gel and electrotransferred to nitrocellulose membrane (Whatman, Protran BA83, Sigma-Aldrich, Rehovot, Israel). The blots were blocked with 5% skim milk and then probed with mouse polyclonal-EhTGT antibody (1:1000) [12] for 16 h at 4 °C. Next, the blots were washed, probed with secondary antibody (Jackson ImmunoResearch, Enco, Petach Tikvah, Israel) at room temperature for 1 h, and developed using enhanced chemiluminescence reagent (Advansta, Bioconsult, Jerusalem, Israel).

### 2.17. Statistical Analysis

Statistical analysis and graphs were performed using Prism 6.02 (GraphPad Software, San Diego, CA, USA). Data are given as mean ± standard error of the mean (SEM) of 2–3 biological replicates. Unless mentioned otherwise, significance was tested by Student’s *t*-test.

## 3. Results and Discussion

### 3.1. Salvage of Queuine from Q and from E. coli K12 by E. histolytica

**Figure 1:**
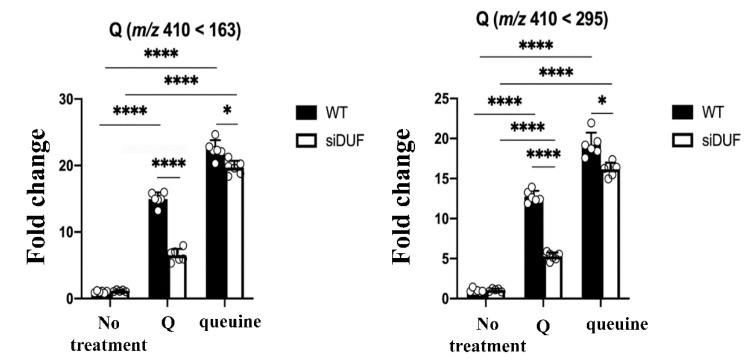
Q level change determined by LC-MS/MS upon trophozoites cultivated with queuine or Q. The fold change is relative to wild type strain without any additive treatment. Data is from two biological replicates, each with three technical replicates. * indicates *p* value < 0.05, **** indicates *p* value < 0.0001, which were determined by one-way ANOVA.

In order to determine whether the parasite can salvage queuine from Q, *E. histolytica* trophozoites were cultivated in the presence of Q, and the Q-tRNA level was determined by LC–MS (Figure 1).

In agreement with our previous data [12], we found that queuine is uptaken by *E. histolytica* trophozoites and incorporated into tRNA (Figure 1). When trophozoites were grown with Q, the level of Q-tRNA was significantly higher than the level in trophozoites grown without Q. These results indicate that a transporter(s) mediates the uptake of Q or queuine inside the parasite. Recently, a queuine transporter, ypdP, has been identified in the pathogenic bacteria *Chlamydia trachomatis* ypdP [6]. However, we did not find a *C. trachomatis* ypdP homolog in *E. histolytica* by using BlastP [23]. Mediated transport of nucleoside in *E. histolytica* has been previously demonstrated [24]**,** and a number of transmembrane proteins have been identified [25]. Parasitic protists possess nucleoside and nucleobase transporters that belong to the equilibrative nucleoside transporter (ENT) family, which has eleven membrane-spanning domains and occurs in animals and plants alike [26]. Four members of the ENT family, namely, (EHI_110730, EHI_017040, EHI_169580, and EHI_142150) have been identified among *E. histolytica* transmembrane proteins [25]**,** and their roles in the transport of queuine and/or Q are currently studied.

**Figure 2:**
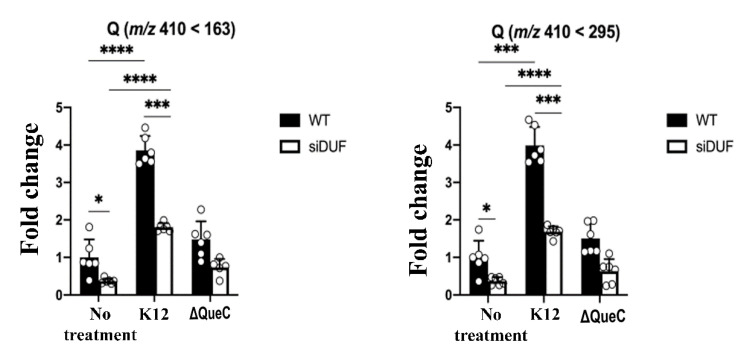
Q level change upon trophozoites cultured with *E. coli* K12 vs. *E. coli* ΔQueC mutants. The fold change is relative to wild-type strain without any additive treatment. Data are from two biological replicates, each with three technical replicates. * indicates *p* value < 0.05, *** indicates *p* value < 0.001, **** indicates *p* value < 0.0001, which were determined by one-way ANOVA.

In the large intestine, *E. histolytica* trophozoites feed on bacteria [27]. Consequently, we hypothesized that the parasite could salvage queuine directly from ingested bacteria. The hypothesis was tested by feeding *E. histolytica* trophozoites with *E. coli* K12, which served as a Q-donor bacteria [28]. According to LC-MS/MS data, trophozoites can salvage queuine from *E. coli* K12 (Figure 2). We confirmed that *E. coli* K12 is the source of queuine as *E. histolytica* trophozoites were unable to salvage queuine from *E. coli* ∆QueC, a mutant that is unable to synthesize queuine [29] (Figure 2). 

**Figure 3:**
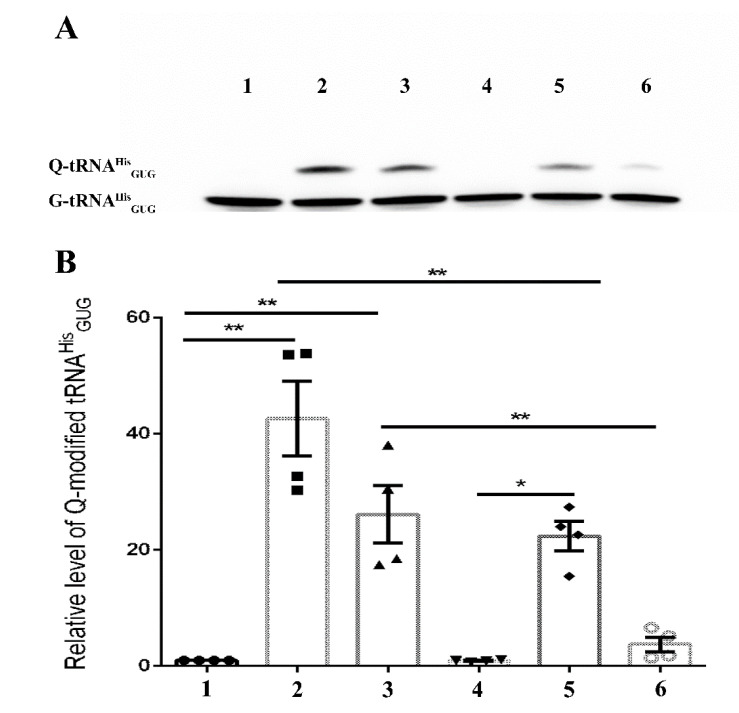
APB Northern blot analysis of tRNA^His^_GUG_ in control (WT) and siEhDUF2419 trophozoites following cultivation with queuine or Q. Control (WT) or siEhDUF2419 trophozoites were cultivated in the presence of 0.1 µM queuine or Q for 3 days. (1) WT trophozoites (2) queuine-treated WT trophozoites (3) Q-treated WT trophozoites (4) siEhDUF2419 trophozoites (5) queuine-treated siEhDUF2419 trophozoites (6) Q-treated siEhDUF2419 trophozoites. Data are from two biological replicates, each with two technical replicates. * indicates *p* value < 0.05, ** indicates *p* value < 0.01. (**A**) APB analysis (**B**) quantitative analysis of relative levels of Q-tRNA^His^_GUG_.

**Figure 4:**
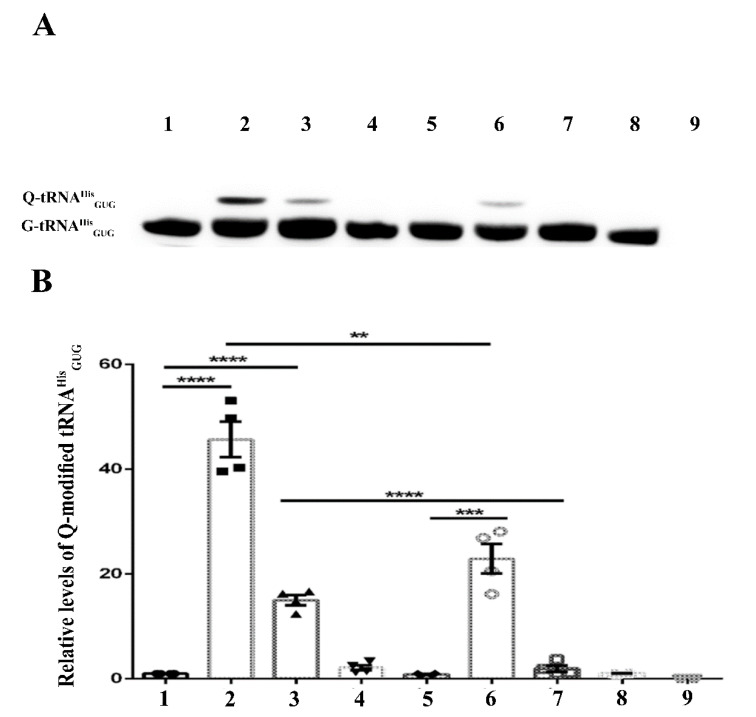
APB Northern blot analysis of tRNA^His^_GUG_ in control (WT) and siEhDUF2419 trophozoites that were co-cultivated with *E. coli* K12 or *E. coli* ∆QueC. Control (WT) and siEhDUF2419 trophozoites were cultivated in the presence of *E. coli* K12 or *E. coli* ∆QueC for 7 days (ration of 1 trophozoite:1000 bacteria). (1) WT trophozoites (2) queuine-treated WT trophozoites (3) WT trophozoites that were cultivated with *E. coli* K12 (4) WT trophozoites that were cultivated with *E. coli* ∆QueC (5) siEhDUF2419 trophozoites (6) queuine-treated siEhDUF2419 trophozoites (7) siEhDUF2419 trophozoites that were cultivated with *E. coli* K12 (8) siEhDUF2419 trophozoites that were cultivated with *E. coli* ∆QueC (9) *E. coli* K12 RNA. Data are from two biological replicates, each with two technical replicates. ** indicates *p* value < 0.01, *** indicates *p* value < 0.001, **** indicates *p* value < 0.0001. (**A**) APB analysis (**B**) Quantitative analysis of relative levels of Q-tRNA^His^_GUG_.

The conclusions drawn from the LC-MS data are further supported by studying the level of Q-tRNA^His^_GUG_ by APB polyacrylamide gel analysis (Figure 3 and Figure 4).

The mammalian gut is crowded with microorganisms fighting for nutrients and survival [30]. Competition for Q also occurs in the gut, and recently, two Q salvage pathways have been characterized by pathogenic and commensal bacteria [6]. *E. histolytica* will be exposed to this Q competition inside the human gut. Data shown in Figure 3 and Figure 4 indicate that the parasite has an advantage over its prokaryotic competitors because it phagocytoses bacteria, the main source of Q. Some bacteria such as *Lactobacillus ruminus* are preferred as a nutritional source by *E. histolytica* over other gut bacteria [27]. There is a possibility that *L. ruminus* may offer the parasite an important source of Q. *L. ruminus* encodes a TGT enzyme in its genome (WP_014073827.1), supporting this hypothesis. 

### 3.2. Characterization of EhDUF2419 as an Enzyme That Salvages Queuine from Q in E. histolytica

The ability of *E. histolytica* to salvage queuine from Q or *E. coli* suggests the presence of an active queuine-salvaging pathway in the parasite. We hypothesize that DUF2419 is involved in this pathway. According to annotations of *E. histolytica*’s genome, there is a homolog of *S. pombe* DUF2419 (accession number Q9HDZ9) in *E. histolytica*, namely, EhDUF2419 (EHI_098190/XP_653631.1). The EhDUF2419 gene is highly homologous to *S. pombe* DUF2419 (query cover 97%; E value 1E**^−^**^28^; percentage identity 27.1%). 

**Figure 5:**
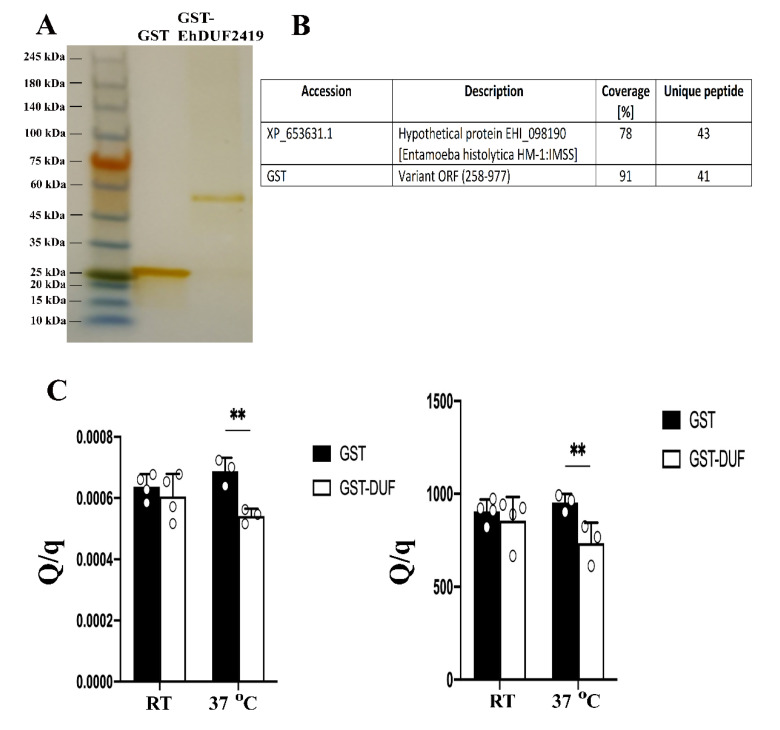
Biochemical characterization of EhDUF2419. (**A**). In total, 10 μg of recombinant GST and GST-tagged EhDUF2419 proteins were resolved on 12% SDS gel and stain with silver staining using Pierce Silver Stain Kit according to the manufacturer’s instructions. (**B**). Confirmation by MS analysis of the nature of GST-EhDUF2419 protein. (**C**). In vitro activity of DUF2419 in Q hydrolysis into queuine. Left panel: ratio of Q UV signal and queuine MS signal; right panel. Ratio of Q MS signal (*m*/*z* 410 > 295) and queuine MS signal. Data are from two biological replicates, each with two technical replicates. ** indicated *p* value < 0.01.

As a first step in the biochemical characterization of EhDUF2419, EhDUF2419 was expressed as a recombinant GST-tagged protein in *E. coli*. We achieved a level of expression of 100 µg of GST-EhDUF2419 per 100 ml of *E. coli* culture. SDS-PAGE analysis followed by silver staining shows a single 62 kDa band, which corresponds to the expected molecular weight for GST-EhDUF2419 (Figure 5A). MS analysis confirmed that the 62 kDa band was GST-EhDUF2419 (Figure 5B). The next step was to test the ability of EhDUF2419 to catalyze the formation of queuine from Q. GST-EhDUF2419 was incubated overnight with Q at room temperature or at 37 °C and the formation of queuine was determined by LC-MS. When GST-EhDUF2419 was incubated in the presence of Q, significant levels of queuine were detected following incubation at 37 °C (Figure 5C). In contrast, no queuine was detected when Q was incubated with GST (Figure 5C). According to these data, EhDUF2419 catalyzes the formation of queuine from Q. The conversion of Q to queuine is not completed. It is possible that the reaction conditions used here are not optimal or that EhDUF2419 is expressed in *E. coli* is not fully functional. It is also possible that Q is not the preferential substrate but rather 5′-QMP, as previously suggested by Gunduz and Katze [13].

**Figure 6:**
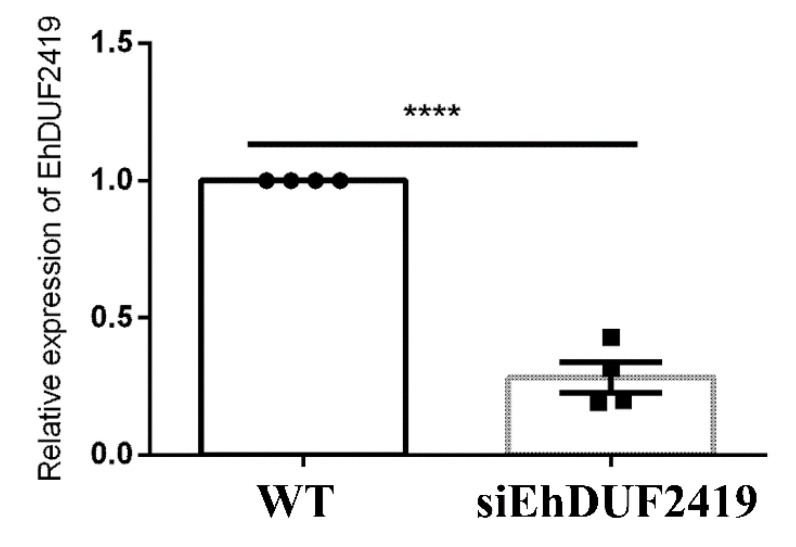
EhDUD2419 expression levels in *E. histolytica* trophozoites. The relative fold change of EhDUD2419 expression in control (WT) and siEhDUF2419 trophozoites was calculated using the 2^^(−ΔΔCt)^ method [21]. Data are from two biological replicates, each with two technical replicates. **** indicates *p* value < 0.0001.

In order to confirm the role of EhDUF2419 as a queuine-salvaging enzyme, we silenced its expression using antisense small RNAs [31], a method previously used to silence the expression of EhQTRT1 [12]. In this method, a gene-coding region to which large numbers of antisense small RNAs map is used as a ‘trigger’ to silence the gene fused to it. Silencing of EhDUF2419 expression was confirmed by qRT-PCR (Figure 6) of EhDUF2419-silenced trophozoites.

Next, we tested the ability of EhDUF2419-silenced trophozoites to salvage queuine from Q or *E. coli* K12. LC-MS indicates that the level of Q-tRNA is strongly reduced in siEhDUF2419 trophozoites that were grown with Q or with *E. coli* K12. However, siEhDUF2419 trophozoites cultivated in the presence of queuine were still able to form Q-tRNAs (Figure 1, Figure 2, Figure 3 and Figure 4). Interestingly, the level of Q-tRNA in siEhDUF2419 trophozoites that were grown with queuine was lower than the level of Q-tRNA in control trophozoites cultivated with queuine (Figure 1). This result suggests that EhDUF2419 and EhTGT are connected. An examination of Q-tRNA^His^_GUG_ levels by APB polyacrylamide gel analysis also supports these conclusions drawn from the LC-MS/MS data (Figure 3).

**Figure 7:**
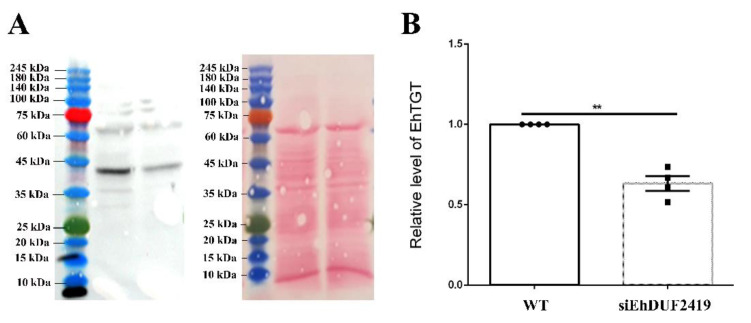
EhTGT level in control (WT) and siEhDUF2419 trophozoites. (**A**). Western blotting was performed on total protein extracts that were prepared from WT *E. histolytica* trophozoites (WT) and siEhDUF2419 trophozoites. The proteins were separated on 12% SDS-PAGE gels and analyzed by Western blotting using a homemade EhTGT antibody (1:1000) [12]. (**B**). Ponceau staining of the membrane before its incubation with EhTGT antibody. The level of EhTGT was normalized according to the total protein amount in each lane as seen by ponceau staining. Data are from two biological replicates, each with two technical replicates. ** indicates *p* value *<* 0.01.

As a first step to understanding how EhDUF2419 and EhTGT are connected, the levels of EhTGT expression in control and siEhDUF2419 trophozoites were determined by WB analysis. We observed that the EhTGT level in siEhDUF2419 trophozoites is 30% less than in control trophozoites (Figure 7). 

In this study, we observed that when the expression of EhDUF2419 is downregulated, the expression of EhTGT is also down. It is possible that EhDUF2419 acts as a transcription factor and regulates EhTGT expression. However, except for homology with DNA glycosidases, no other function can be deduced from the DUF2419 sequence [15]. EhDUF2419 may be needed for EhTGT activity, but we have already demonstrated that EhTGT is catalytically active without any additional proteins added to the reaction [12]. Finally, EhDUF2419 can also regulate the stability of EhTGT in the parasite. This hypothesis is currently under investigation. Although the link between EhDUF2419 and EhTGT needs more investigation to be understood, the reduction of EhTGT level in siEhDUF2419 trophozoites can explain why less Q-tRNA^His^_GUG_ was observed in siEhDUF2419 trophozoites cultivated in the presence of queuine. We previously reported that the level of Q-tRNAs in the parasite correlates with OS resistance [12]. As less Q-tRNA^His^_GUG_ is formed in siEhDUF2419 trophozoites cultivated in the presence of queuine, the resistance to OS is, therefore, lower than in control trophozoites cultivated in the presence of queuine. 

### 3.3. Phenotypical Characterization of siEhDUF2419 Trophozoites

**Figure 8:**
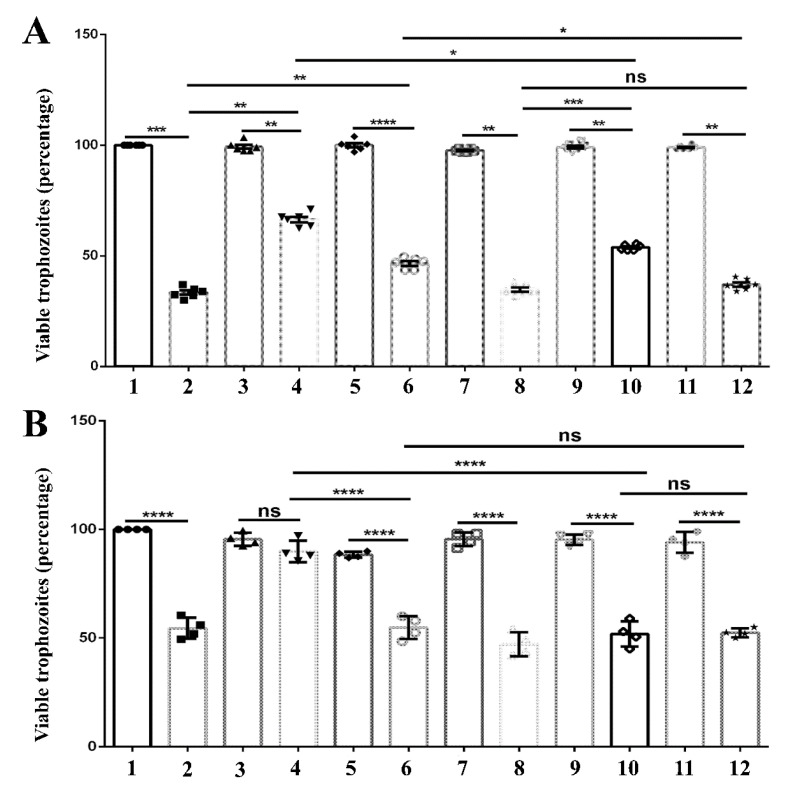
Resistance to OS in queuine/Q-treated *E. histolytica* trophozoites. The 1 × 10^6^ control/siEhDUF2419 trophozoites that grow in the presence of 0.1 µM queuine or Q were exposed to 2.5 mM H_2_O_2_ for 30 min. (**A**). (1) Control trophozoites. (2) Control trophozoites + OS. (3) queuine-treated control trophozoites. (4) queuine-treated control trophozoites + OS. (5) Q-treated control trophozoites. (6) Q-treated control trophozoites + OS. (7) siEhDUF2419 trophozoites. (8) siEhDUF2419 trophozoites + OS. (9) queuine-treated siEhDUF2419 trophozoites. (10) queuine-treated siEhDUF2419 trophozoites + OS. (11) Q-treated siEhDUF2419 trophozoites. (12) Q-treated siEhDUF2419 trophozoites + OS. Data are from three biological replicates, each with two technical replicates. * indicates *p* value < 0.05. ** indicates *p* value < 0.01. *** indicates *p* value < 0.001. **** indicates *p* value < 0.0001. (**B**). (1) Control trophozoites. (2) Control trophozoites + OS. (3) Control trophozoites that were cultivated with *E. coli* K12. (4) Control trophozoites that were cultivated with *E. coli* K12 + OS. (5) Control trophozoites that were cultivated with *E. coli* ΔQueC. (6) Control trophozoites that were cultivated with *E. coli* ΔQueC + OS. (7) siEhDUF2419 trophozoites. (8) siEhDUF2419 trophozoites + OS. (9) siEhDUF2419 trophozoites that were cultivated with *E. coli* K12. (10) siEhDUF2419 trophozoites that were cultivated with *E. coli* K12 + OS. (11) siEhDUF2419 trophozoites that were cultivated with *E. coli* ΔQueC. (12) siEhDUF2419 trophozoites that were cultivated with *E. coli* ΔQueC +OS. Data are from two biological replicates, each with two technical replicates. * indicates *p* value < 0.05. ** indicates *p* value < 0.01. *** indicates *p* value < 0.001. **** indicates *p* value < 0.0001.

In this study, we examined the effect of silencing EhDUF2419 expression on parasite growth. Our results indicate that this had no effect on the growth of the parasite (Supplementary Figure S1). Our previous work has shown that queuine protects the parasite against OS by triggering the expression of genes associated with stress response [12]. Here, we have investigated the response of siEhDUF2419 trophozoites exposed to queuine or Q. We observed that queuine but not Q protects siEhDUF2419 trophozoites against OS (Figure 8A). We also observed that *E. coli* K12 but not *E. coli* ∆QueC protects the parasite against OS (Figure 8B).

## 4. Conclusions

**Figure 9:**
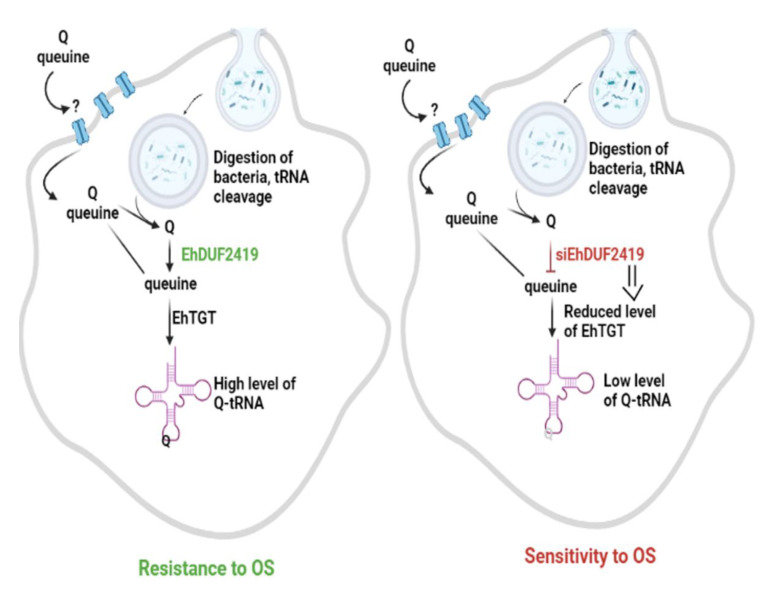
Queuine salvaging in *E. histolytica. E. histolytica* uptakes queuine or Q from its environment but the nature of the transporter(s) is still unknown. EhDUF2419 salvages queuine from Q or from phagocytosed *E. coli* cells. A study is underway to determine whether queuine salvaging occurs inside or outside of phagosomes. Next, EhTGT incorporates queuine into the wobble position of tRNA^His^, tRNA^Asp^, tRNA^Asn^ and tRNA^Tyr^, which induces the expression of stress response proteins [12]. In contrast, silenced-EhDUF2419 trophozoites are not capable to salvage queuine from Q. A low level of EhDUF2419 expression impairs the level of EhTGT expression by a yet undetermined mechanism. The combination of both events (silencing of EhDUF2419 expression and low level of EhTGT expression) leads to low level of Q-tRNAs formed in the parasite and more sensitivity to OS. (Created with BioRender.com on 21 July 2022).

In organisms that presumably obtain Q from degraded bacteria tRNA material, DUF2419 has been shown to serve as an enzyme that salvages queuine from Q [15]. This study, whose main results are summarized in Figure 9, demonstrates the requirement for DUF2419 in order for *E. histolytica* to salvage queuine from phagocytosed bacteria. Obtaining queuine at the source may help the parasite compete more efficiently for this nutrient with gut bacteria that have been shown to salvage queuine [6]. We have recently reviewed a number of bacterial metabolites that influence the biology of the parasite, including the resistance to OS [32]. Q and queuine, which protect *E. histolytica* against OS (this work and [12]), represent additional bacterial metabolites that may help the parasite to survive in the large intestine when dysanerobiosis occurs as a result of inflammatory conditions [33] or from dysbiosis [34]. The targeting of the queuine salvage pathway identified in this study may affect the parasite’s ability to sustain OS during its life cycle in the host and, therefore, its ability to spread.

## Figures and Tables

**Table 1:** A list of primers used to amplify EhDUF2419 and rDNA.

Primer Name	Primer Sequence	Direction	Used for
5′ BamHI EhDUF2419	GGATCCATGTGTGAATATGTTCGTTGG	Sense	pGEX-EhDUF2419 vector
3′ EhDUF2419	ATAAAAAATGGTTTGTGTTCGGTGG	Anti-sense	pGEX-EhDUF2419 vector
5′ EhDUF2419 set 3	CACCCTGAAGTTTTTGAGCC	Sense	qPCR
3′ EhDUF2419 set 3	GGTTGAATCTCTAAACCCAGG	Anti-sense	qPCR
5′ BglII EhDUF2419	AGATCTATGTGTGAATATGTTCGTTGGA	Sense	siEhDUF2419 vector
3′ XhoI EhDUF2419	CTCGAGATAAAAAATGGTTTGTGTTCGGTGG	Anti-sense	siEhDUF2419 vector
rDNA5′	TCAAAAAGCAACGTCGCTA	Sense	qPCR
rDNA3′	AGCCCGTAAGGTGATTTCT	Anti-sense	qPCR

## Data Availability

Not applicable.

## Supplementary Materials

The following supporting information can be downloaded at: https://www.mdpi.com/article/10.3390/cells11162509/s1, Figure S1: Growth rate of *E. histolytica* siEhDUF2419 trophozoites.
