# Supplementary material for: Queuine Salvaging in the Human Parasite Entamoeba histolytica
Source: Cells. 2022 Aug 12;11(16):2509. doi: 10.3390/cells11162509 (PMC9406330; doi:10.3390/cells11162509)
Supplement: Supplementary file 1 [file cells-11-02509-s001.zip › cells-1817745-supplementary.pdf]

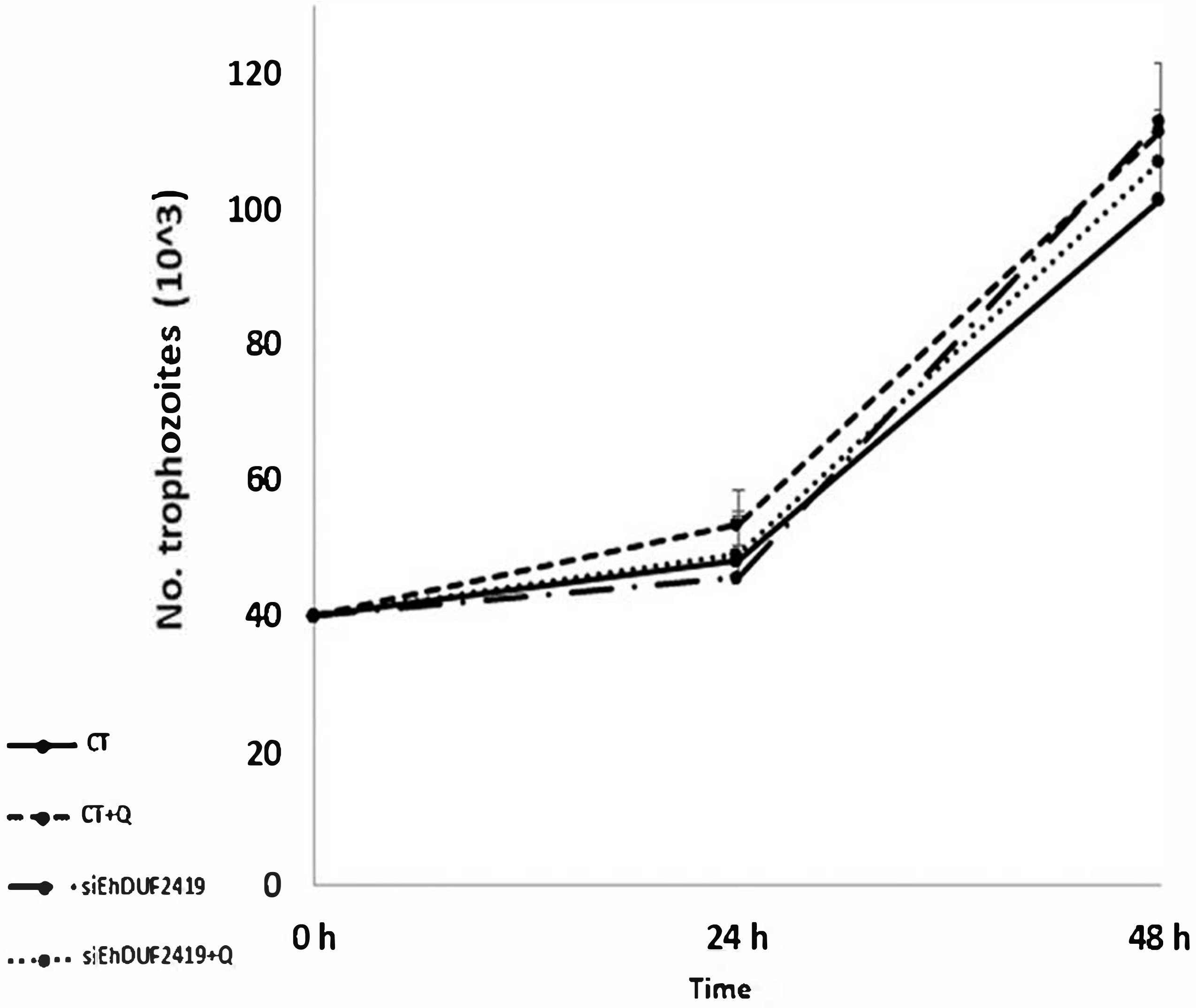

Supplementary Figure S1. Growth rate of *E. histolytica* siEhDUF2419 trophozoites.

$40*10^3$ control trophozoites and siEhDUF2419 trophozoites were grown for 24 hours or 48 hours in 15-mL tube with/without 0.1μM queuosine. The number of viable trophozoites was determined by eosin dye exclusion. The data represent two independent experiment that were repeated twice. p-value > 0.05 by an unpaired Student *t* test.
